# Respiratory Sinus Arrhythmia, Effortful Control, and Child Social Anxiety Symptoms

**DOI:** 10.1007/s10802-024-01202-z

**Published:** 2024-04-26

**Authors:** Elizabeth J. Kiel, Elizabeth M. Aaron

**Affiliations:** Department of Psychology, Miami University, 100 Psychology Building, 45056 Oxford, OH USA

**Keywords:** Children, Anxiety, Temperament, Psychophysiology

## Abstract

Emotion dysregulation is implicated in child social anxiety and its etiology. Child emotion dysregulation has been studied via physiological indicators (e.g., respiratory sinus arrhythmia [RSA]) and behavioral indicators (e.g., effortful control). Previous work suggests that physiological indicators of regulation may predict outcomes in a non-linear manner and must be considered within the context of other intrapersonal factors, perhaps including effortful control. To this end, the current study tested effortful control as a moderator of the relation between RSA and child social anxiety, considering both linear and curvilinear patterns and controlling for inhibited temperament, an established predictor of child anxiety. Children (*n* = 119; 44% female) participated when they were 4 years old and entering school age (5 to 7 years). Mothers reported on children’s effortful control (age 4) and social anxiety (school age). Children’s RSA (age 4) was calculated from electrocardiogram data when they were at rest (i.e., baseline RSA) and when they were giving a speech. Results indicated that when children were high in effortful control, lower baseline RSA predicted higher social anxiety symptoms. Tentative evidence emerged for a relation between greater suppression of RSA during the speech compared to baseline and higher social anxiety symptoms when children were low in effortful control. Results support assessing the temperamental domain of effortful control as a contextualizing factor in the relation between psychophysiology and child anxiety outcomes.

**Supplementary Information:**

The online version contains supplementary material available at 10.1007/s10802-024-01202-z.

## Respiratory Sinus Arrhythmia, Effortful Control, and Children’s Social Anxiety Symptoms

Social anxiety has attracted attention among developmental psychopathologists because of its uniquely strong links to emotion-related biological markers and behaviors identifiable in early development (Fox et al., 2023). Emotion dysregulation, characterized by difficulties understanding, accepting, and managing emotions in service of one’s goals (Beauchaine, 2015), is a crucial factor implicated in child social anxiety and its etiology (Cisler et al., 2010). Child emotion dysregulation (or the inverse, emotion regulation) has been studied via physiological indicators (e.g., respiratory sinus arrhythmia [RSA], an index of the parasympathetic nervous system) and behavioral indicators (e.g., effortful control, a temperamental dimension reflecting higher-level, top-down regulation). Both RSA and effortful control are implicated in anxiety development (Monk et al., 2001; Raines et al., 2019; Sharma et al., 2011; White et al., 2011). However, it has been suggested that physiological indicators of regulation such as RSA must be considered within the context of other intrapersonal factors (Mendes, 2016). Because temperament is, broadly, an organizing construct, it may be that psychophysiology has different implications for children of varying temperamentally-based effortful control. Eisenberg’s model of emotion-related regulation (Eisenberg et al., 2014; Eisenberg & Morris, 2002) guided our examination of both RSA and parent-reported effortful control as relevant to emotion dysregulation in relation to social anxiety. Importantly, Eisenberg’s model predicts that indicators of voluntary control can be differentiated from reactive control, which inhibits behavior. It is well established that child inhibited temperament, individual differences in reactivity to novelty and uncertainty, predicts child anxiety (Fox et al., 2023; Kagan et al., 1984). Thus, the current study tested moderation over and above inhibited temperament.

### Respiratory Sinus Arrhythmia

RSA, a measure of the variation in the time between (i.e., interbeat interval of) a person’s heart beats at the rate of respiration, is an index of the parasympathetic nervous system, which is responsible for regulating the body after episodes of reactivity (Porges, 1995). RSA is one of several measures of heart rate variability (HRV). Root mean square successive difference (RMSSD) is another measure of HRV that some research has suggested is less affected by changes in respiration than RSA (Shaffer & Ginsberg, 2017). However, it has been noted that RMSSD is still impacted by changes in respiration rate as well as by heart period, and thus should be interpreted with caution (Berntson et al., 2005). Frequency-derived measures of HRV, such as RSA, and statistically-derived measures, such as RMSSD, are similarly correlated with top-down self-regulation (Holzman & Bridgett, 2017). The majority of developmental studies focus on RSA (Hastings & Kahle, 2019); we therefore did the same.

When measured at rest, RSA is considered an indicator of one’s capacity for self-regulation, including emotion regulation specifically, with lower values indicating lower regulation, or greater dysregulation (Holzman & Bridgett, 2017; Porges, 1995). Eisenberg and Morris’s (2002) heuristic model of emotion-related regulation predicted that indicators of regulation, like RSA, would have a negative relation to internalizing outcomes like social anxiety. Indeed, lower resting RSA has been found to relate to higher anxiety (Sharma et al., 2011). Other research indicates a curvilinear relation, such that both very low and very high resting RSA related to higher internalizing problems (Ugarte et al., 2021).

RSA reactivity, or the change in RSA from rest to a challenging or threatening task, has been posited to reflect an individual’s acute modulation of their regulation in response to environmental demands (Porges, 1995). Greater RSA suppression (a decrease in RSA from resting to task) indicates withdrawal of parasympathetic control and is associated with a “threat” response; greater RSA augmentation (increase in RSA from rest to task) indicates increased parasympathetic control and engagement with challenge (Hastings et al., 2008). There is mixed evidence as to whether greater or lesser change in RSA is more adaptive or specifically related to anxiety (Alkozei et al., 2015; Monk et al., 2001; Trent et al., 2020). However, research using a speech task, which is highly relevant to child social anxiety, showed that temperamental shyness (a potential precursor to social anxiety) related to the “threat” response of greater RSA suppression (Poole & Schmidt, 2021). Thus, existing literature suggests that low RSA at rest and greater RSA suppression during a social stressor serve as endophenotypes of emotion regulation difficulties that are implicated in anxiety-based psychopathology (Lenzenweger, 2013).

A physiological response may reflect different emotion and regulatory processes depending on the situation, the developmental stage, and the individual’s other capacities and characteristics (Beauchaine, 2001; Mendes, 2016). Individuals with lower resting RSA may be more likely to perceive situations as threatening due to having less physiological capacity to self-regulate. However, the unified theory of development suggests that many internal and external forces contextualize child development (Sameroff, 2009), including how a child interprets situations. Further, when considering RSA reactivity to a new situation, it has been posited that the manner in which an individual interprets a situation determines one’s regulatory response. If an individual perceives a situation as one that they have the capacity to manage effectively (i.e., their resources outweigh the demands of the situation), their RSA may increase (augment) in order to approach the challenging situation. Conversely, if an individual perceives that same situation as one that is unmanageable (i.e., the demands outweigh their resources), their RSA may decrease, resulting in suppression, in order to conserve energy and withdraw from the threatening situation (Mendes, 2016). These ideas are born out in experimental studies that manipulate the demands of the situation, as well as in studies examining RSA responses in individuals varying in internal characteristics relevant to coping resources (Mendes, 2016; Poole & Schmidt, 2021). In children, one internal factor that may serve an important organizational role in the consequences of psychophysiological dysregulation is effortful control.

### Effortful Control

Temperament represents biologically based individual differences in how one is predisposed to respond to the environment and incorporates components of both reactivity and regulation (Shiner et al., 2012). Temperament can be considered an organizer of individuals’ development and environmental responses. Effortful control is a temperamental dimension defined by higher-level regulatory capacities for inhibiting dominant responses, waiting, planning, and controlling one’s attention to meet the demands of the situation (Eisenberg et al., 2014; Putnam & Rothbart, 2006). Importantly, Eisenberg and colleagues differentiate effortful control from reactive control, which inhibits behavior but does not assist in (and actively undermines) the volitional control of emotional reactivity (Eisenberg et al., 2014; Eisenberg & Morris, 2002). Effortful control shares many characteristics with executive functioning, defined as one’s higher-order control over attention, cognition, and behavior. Effortful control and executive functioning have common underlying features, such as attentional and behavioral control. However, executive functioning develops later in life, changes over time, and includes complex thought processes and goal-directed behaviors whereas effortful control is an early-developing and stable dimension that does not include advanced cognitive skills (Liew, 2012; Nigg, 2017). Nonetheless, both effortful control and executive function reflect an individual’s capacity for top-down self-monitoring and regulation (Eisenberg et al., 2014). In sum, effortful control organizes the way in which individuals self-regulate through top-down processes, with higher effortful control reflecting a greater amount of top-down regulatory capacity.

Effortful control’s role in anxiety development may be direct, but there is increasing support for its consideration as a moderator or contextualizing factor. Eisenberg’s model of effortful control predicts a direct negative relation to internalizing outcomes like anxiety, and some empirical studies support this. Research has indicated that effortful control negatively relates to child anxiety and buffers against the development of anxiety in children with other temperamental risk factors (Affrunti & Woodruff-Borden, 2015; Niditch & Varela, 2018; Raines et al., 2019). Other research has found that effortful control does not directly relate to anxiety, but instead interacts with other risk factors in the prediction child anxiety as either a protective (Gulley et al., 2016) or risk (White et al., 2011) contextualizing factor. Previous work has found an interaction such that higher inhibitory control, the aspect of effortful control aligned with holding back a response, predicted fewer externalizing problems within the context of greater RSA augmentation (Kahle et al., 2018). Although not specific to social anxiety, this work does suggest that effortful control may contextualize relations between RSA and child outcomes.

The moderating role of effortful control in the relation between RSA and child anxiety, specifically, has yet to be studied. Eisenberg and Morris’s (2002) heuristic model, as well as other models of self-regulation (e.g., Posner &Rothbart, 2000), suggests that risk for internalizing problems such as anxiety would emerge at low effortful control, whereas optimally regulated children benefit from the systemic organization of high effortful control. Thus, it is in this context of low effortful control that we may expect the negative relation between RSA and anxiety to be the strongest, revealing that children with low RSA and low effortful control have the highest anxiety. For children with high effortful control who may feel more prepared to manage difficult tasks even when they have poorer physiological supports for self-regulation, the relation would weaken. It should also be acknowledged that the moderation could occur in another direction: it may be that children with high effortful control and low RSA become more distressed at their inability to regulate their physiological response due to their typical capacity for behavioral and cognitive regulation. This could represent discordance among regulatory systems, resulting in higher negative emotionality that poses risk for anxiety outcomes (Hassan et al., 2018). To the extent that reactive control is imperfectly parsed from effortful control, this could also reflect a rigid “overcontrol” that behaviorally manifests as regulation to parent-reporters but belies an underlying inflexibility. Inhibitory control, specifically, has been conceptualized as a moderator that increases, rather than decreases, the strength of a developmental trajectory to social anxiety (Fox et al., 2023). Thus, it remains to be determined how effortful control contextualizes the relation between RSA and anxiety, though theory would support a negative relation between RSA and anxiety at low effortful control. A strong test of this relation would also consider the role of inhibited temperament.

### Inhibited Temperament

Inhibited temperament, typically studied in early childhood, reflects an individual’s propensity towards reactivity to novelty and uncertainty, which is manifested as withdrawn, hesitant, and fearful behavior (Fox et al., 2023; Kagan et al., 1984). These behaviors are consistent with those often demonstrated by children with anxiety. Further, the physiological profile of children with inhibited temperament suggests that this temperamental dimension may capture some of the genetic loading for anxiety. Children with highly inhibited temperament have an endophenotype in which the amygdala, which is central to the neural circuitry of anxiety (Davis, 1992), has a low threshold for activation, thus setting off multiple systems that inhibit approach (Schwartz et al., 2003). Indeed, inhibited temperament is one of our most robust predictors of child social anxiety symptoms (Fox et al., 2023; Pérez-Edgar & Guyer, 2014). Thus, it is important to understand whether RSA and effortful control play a role in child anxiety development unique from the well-established path of risk from inhibited temperament. Including inhibited temperament also allows for the prediction of increased risk above and beyond pre-existing levels. Moreover, Eisenberg and Morris’s (2002) heuristic model of emotion-related regulation aligns inhibited behavior with reactive control and notes the importance of studying both effortful and reactive control simultaneously to identify their unique contributions to outcomes.

### Current Study

Previous research has demonstrated a predictive relation between RSA and child anxiety, but it is increasingly clear that this relation may be nonlinear and contextualized. Eisenberg and colleagues’ (Eisenberg et al., 2014; Eisenberg & Morris, 2002) conceptualization of emotion-related regulation provides a useful model from which to derive predictions for how its indicators, in inverse, represent dysregulation and predict social anxiety. Thus, the current study investigated effortful control as a moderator of the longitudinal relation between RSA and child social anxiety, controlling for child inhibited temperament. Based on existing models of self-regulation (Eisenberg & Morris, 2002; Posner & Rothbart, 2000), we hypothesized that the expected negative relation between resting RSA and social anxiety would be especially strong at low effortful control. In other words, when there is low top-down regulation, we expected lower capacity for biological regulation to predict higher social anxiety symptoms. Based on a recent study showing that RSA suppression during social performance related to shyness (Poole & Schmidt, 2021), we also hypothesized that greater RSA suppression would predict social anxiety symptoms, also only when effortful control was low. In other words, when children were reported to have lower top-down regulation, we expected greater “threat” response to the speech task to predict greater social anxiety.

## Method

### Participants

As part of a larger longitudinal study (Kiel et al., 2021), 119 children (52 assigned female at birth) participated in a laboratory visit at approximately 4-years-old (*M* = 52.01 months, *SD* = 3.48 months). Most children (*n* = 107) had participated in previous waves of the study (ages 1, 2, and 3 years); 12 were newly recruited at the age 4 wave. Across waves, families were recruited via direct mailings to families posting birth announcements in local newspapers; via flyers at childcare centers, pediatricians’ offices, and other local establishments; and in-person at local events and a Women, Infants, and Children’s office. Children participated with their mothers, who reported their own and their children’s racial backgrounds, respectively, to be Asian/Asian American or Pacific Islander (1.7%, 1.7%), Black/African American (2.5%, 3.4%), multiracial (0%, 10.9%), White/European American (95%, 83.2%) or an identity not listed on the form (0.8%, 0.8%). Mothers reported themselves and their children, respectively, to be predominantly non-Hispanic/Latinx (99.2%, 97.5%). Maternal education was, on average, a college degree (*M* = 15.99, *SD* = 2.55 years); 30% of the sample reported less than a college degree. Family income ranged from <$15K/year to >$100K/year, with the average (both mean and median) being in the range of $61–70 K/year (24% reported <$40K/year). A follow-up assessment occurred at early school age, when children were 5 to 7 years old (*M* = 5.48, *SD* = 0.49).

### Procedure

All procedures were approved by the Miami University Institutional Review Board (protocol #00248r, 01026r, 01245r). The study was not pre-registered.

Although the primary focus of the current study is between the age 4 and school age assessments, inhibited temperament was derived from earlier assessments. At child ages 1, 2, and 3 years, mother-child dyads had participated in a laboratory visit. Mothers completed written consent on behalf of themselves and their child. The current study uses data only from a battery of tasks involved in the assessment of inhibited temperament (Fox et al., 2001). A primary experimenter (E1) showed the mother and child into a large room containing a variety of toys and a chair for the mother. E1 instructed the child to “play however you like” and instructed the mother to sit in the chair. E1 then left the room. After 5 min, E1 returned and asked the mother and child to clean up the toys into a bin. When this was complete, E1 removed the bin and left the room. Shortly after, a different experimenter (E2) entered the room and sat in the corner for 2 min without engaging the child, beginning the “Stranger” portion of the task. They then took a toy dump truck and several soft blocks out of a bin, playing with them for 2 min and prompting the child to play at designated times if they had not already approached. E2 then put the dump truck and blocks away and brought out a remote-controlled robot, beginning the “Robot” segment. The robot moved and made noises seemingly randomly for 1 min. E2 prompted the child to approach the robot if they had not already at designated times. Then, E2 put the robot away and brought out an expandable tunnel, beginning the “Tunnel” task, prompting the child to crawl through. If the child did not crawl into the tunnel, E2 put a block inside the tunnel and asked the child to retrieve it. E2 prompted the child an additional time, then said goodbye and left the room.

When the child was approximately 4-years-old, each mother-child dyad came to the laboratory for 1.5 h. An E1 oriented them to the lab, explained procedures, and obtained written consent from the mother on behalf of herself and the child. The mother completed a variety of questionnaires, including a survey of child temperament, used for the assessment of effortful control. After several procedures irrelevant to the current study, E1 introduced a BIOPAC ambulatory electrocardiogram (ECG) and respiration monitor (Biopac Systems, 2016). E1 allowed the mother and child time to affix electrodes (in a 3-lead [Lead II] formation) and respiration belt to the child according to instructions. The child then sat with the mother quietly for a 5-minute baseline recording. The child continued wearing the monitor for the remainder of the visit, including a speech task. In the speech task, the mother and child were instructed to work together to prepare a speech that the child would give about their last birthday. After 3 min of preparation time, the child was instructed to face a camera and give the speech. The mother was instructed to help as she saw fit. The child was given 3 min to give the speech. The current study used ECG from the 3 min of active speech-giving. The speech task was also coded offline for child behaviors relevant to anxiety.

At the school age follow-up visit, mothers again completed written consent. Mothers reported on their children’s social anxiety symptoms as part of a packet of questionnaires.

For observational procedures (inhibited temperament, speech behavior), unique teams of coders were trained by a primary coder and required to reach inter-rater reliability (intra-class correlation [ICC] > 0.80) before coding independently. The primary coder scored at least 20% of cases throughout coding to assess for coder drift, providing guidance to coders as necessary.

### Measures

#### Inhibited Temperament

From the video-recorded procedures for inhibited temperament (above), children’s behaviors (i.e., play, proximity, vocalizations, freezing, vigilant staring, approach, touch) were coded on measures of latency, frequency, and duration (see Kiel et al., 2021 for full description). The final interrater reliabilities were all above .80 (ICC range =.811–1.00, mean =.951). Based on a principal components analysis (PCA) of all coded behaviors from the age 1 visit, 16 behaviors (duration of proximity to mother while not playing during Stranger, Robot, and Tunnel; frequency of non-distressed vocalizations [reversed] in Stranger; duration of play in proximity of the stranger in Stranger, Robot; duration of freezing in Stranger, Robot; duration of vigilant stare in Stranger, Robot, and Tunnel; latency to approach the stimulus in Robot; latency to touch the stimulus in Stranger, Robot; duration of touching the stimulus in Robot) were identified, explaining 44.65% of the variance. These behaviors were standardized and averaged to create a measure of inhibited temperament. A PCA of these 16 behaviors revealed single components for age 2 behaviors and age 3 behaviors, so the same composite was formed for each wave. Inhibited temperament composites at child ages 1, 2, and 3 years were averaged together as an overall measure of inhibited temperament across infancy and toddlerhood. The composite had good internal consistency at each age (αs =.91-.93).

#### Respiratory Sinus Arrhythmia

Children’s ECG was imported into MindWare HRV Analysis 3.1.5 software (Mindware Technologies, 2014) for cleaning and analysis. The baseline and speech task were each divided into 1-minute segments (Task Force of the European Society of Cardiology and the North American Society of Pacing and Electrophysiology, 1996) and filtered at the standard frequency of child respiration (0.24 to 1.04 Hz). Given a recent recommendation that a frequency band of 0.33 to 0.50 more accurately captures respiration for children the age of our sample (Shader et al., 2018), we provide analyses with RSA scored in this manner (which yielded nearly identical results) in the Supplementary Information (Table S1). Each segment was visually inspected for missing/extraneous beats. Segments missing > 10% of beats were not analyzed. Values were averaged across the 5 min of the baseline (resulting in the final “baseline RSA” variable) and the 3 min of the speech task. The final “speech RSA” variable was the unstandardized residual of a regression of average RSA from the speech task on the final baseline RSA variable. Thus, “speech RSA” reflects change in RSA in the speech from expected values based on the baseline. Although primary analyses focus on RSA, we also extracted RMSSD and derived baseline and speech variables in a similar manner to include in a sensitivity analysis.

#### Effortful Control

Mothers reported their children’s effortful control at the age 4 assessment via the Effortful Control domain of the Child Behavior Questionnaire (CBQ) Short Form, a parent-report measure of child temperament (Putnam & Rothbart, 2006). The Effortful Control domain includes four subscales (inhibitory control, attentional focusing, low intensity pleasure, and perceptual sensitivity) with six items each, for a total of 24 items. An example item is, “Can wait before entering into new activities if they are asked to.” For each item, mothers rated how well the statement describes their child in the previous six months on a scale from 1 (*extremely untrue of your child*) to 7 (*extremely true of your child*). Subscales were averaged to yield a domain score, with higher scores reflecting greater effortful control. The short form of the CBQ demonstrates cross-informant reliability between caregivers and moderate stability over one year (Putnam & Rothbart, 2006). In the current sample, stability from age 4 to the follow-up school age assessment was strong (*r* =.73, *p* <.001). The effortful control domain had adequate internal consistency across all items in the current study (α =.83).

#### Speech Behavior

Coders scored children’s distress and shyness during the speech (one score each for the task). Distress (ICC =.78) was negative facial affect, negatively-valenced vocalizations, and direct comments about feeling negatively and was scored on a 3-point scale (1 = *no distress*, 2 = *one or two subtle displays of low intensity distress*, 3 = *definite or prolonged display[s] of distress*). Shyness comprised withdrawal, fidgeting, physical self-soothing, hiding the face, avoidance, vigilance, and bodily tension. Shyness (ICC =.84) was scored on a 5-point scale (1 = *no shyness*, 2 = *slight/brief displays*, 3 = *moderate/continuing displays*, 4 = *high tension, inactivity, and/or avoidance for most of the task*, 5 = *extreme behaviors and/or total avoidance*).

#### Social Anxiety Symptoms

Mothers reported on children’s social anxiety symptoms at the school age assessment via the Social Anxiety subscale of the Preschool Anxiety Scale (Spence et al., 2001). The Social Anxiety subscale includes 6 items reflecting children’s fears about speaking to peers and adults and the potential for embarrassment in social situations. Parents answer items according to what best describes their child on a scale from 0 (*not at all true*) to 4 (*very often true*). An example item is, “Is scared to ask an adult for help.” Item responses were averaged to create the social anxiety symptoms variable (α =.84). The Preschool Anxiety Scale has convergent validity with the Child Behavior Checklist Internalizing Problems subscale (Spence et al., 2001). Within our sample, 17% of children (*n* = 15) had scores on the social anxiety subscale that indicated elevated symptoms (T-scores of 61 or above). It is estimated that approximately 7% of children in the United States have anxiety concerns (Ghandour et al., 2019), indicating that our sample has sufficient representation of children with elevated anxiety.

## Results

Deidentified data and syntax for primary models are publicly available on Open Science Framework (https://osf.io/hmzrx/?view_only=61c9e91c6b924351a8e413235df10cac).

### Preliminary Analyses

Descriptive statistics are presented in Table 1. Inhibited temperament, effortful control, baseline and speech RSA, and social anxiety reasonably adhered to a normal distribution (all|skew| < 2.00, all|kurtosis| < 3.00). We inspected *p*-values associated with Mahalanobis Distance to determine the existence of multivariate outliers, after using the expectation-maximization algorithm to complete a single imputation of missing values (so that all participants could be included). One outlier emerged (*p* <.001). Primary analyses completed with versus without this participant did not differ in the pattern of significance and yielded nearly identical coefficients; results with the outlier included are reported to maximize sample size and generalizability.

**Table 1:** Descriptive Statistics and Pearson Correlations

Variable	*Mean* (*SD*)	*Range* (*Min*-*Max*)	2	3	4	5	6
1. Inhibited Temperament	0.04 (0.56)	-0.98–1.48	.13	.04	−.07	.06	.33**
2. Age 4 Baseline RSA	5.10 (1.09)	1.45–7.51	--	.86***	.00	.02	−.03
3. Age 4 Speech RSA (raw)	5.04 (1.08)	1.97–8.76		--	.51***	.06	.04
4. Age 4 Speech RSA (residualized)	0.00 (0.55)	-1.23–1.73			--	.01	−.12
5. Age 4 Effortful Control	5.30 (0.58)	2.58–6.42				--	−.02
6. Age 5 Social Anxiety	1.01 (0.81)	0.00–3.33					--

Note. RSA = respiratory sinus arrhythmia. Inhibited Temperament is a composite of z-scored behaviors. Age 4 Speech RSA (residualized) represents the unstandardized residuals from a simple linear regression model in which Age 4 Speech RSA (raw) was regressed on Age 4 Baseline RSA.

**p <.01, ***p <.001.

Bivariate correlations among primary variables are also presented in Table 1. As expected, inhibited temperament predicted social anxiety symptoms. Inter-relations also existed among RSA variables. Child sex, maternal education, household income, child age at the age 4 and school age visit, and maternal age at the birth of the child did not relate to social anxiety symptoms (all *p*s >.05), so they were not included as covariates in primary analyses.

Descriptive statistics for speech behaviors are provided to contextualize primary results. Scores of distress covered the entire 1 to 3 range and, on average, indicated low-intensity to more definite distress (*M* = 1.86, *SD* = 0.84) that differed from “no distress” (*t*[109] = 10.78, *p* <.001, Cohen’s *d* = 1.03). Thirty-two children (26.9%) scored the maximum value indicating definite distress. Shyness scores ranged from 1 to 4, with the average score (*M* = 2.60, *SD* = 0.76) indicating moderate shyness that differed from “no shyness” (*t*[109] = 22.18, *p* <.001, Cohen’s *d* = 2.12). Nine children (7.6%) scored a 4, indicating significant withdrawal, nervousness, and/or avoidance, and 57 children (47.9%) scored a 3, suggesting at least moderate shyness.

### Missing Values

Inhibited temperament was missing for 13 children, 12 of whom joined the study at age 4, after those assessments took place (ages 1 through 3). Effortful control was missing from 11 families. Several children were missing baseline RSA, either because the child or mother refused the procedure (*n* = 17, 1, respectively), because of technical difficulties (*n* = 2), because the data were too messy to be analyzed (*n* = 4), or because the family had moved since joining the study and were only able to complete survey measures by mail (*n* = 5). An additional 14 children removed the ECG recording equipment prior to the speech task, and a few additional children were missing RSA from the speech task because of a technical problem/experimenter error (*n* = 5) or data were too messy (*n* = 1). Finally, social anxiety symptoms were missing either because the family was lost to attrition (*n* = 25) or because the mother only partially completed study measures (*n* = 6). Overall, 22.52% of values on primary variables were missing.

We compared participants with versus without missing values on primary study measures in regards to other primary measures as well as demographic characteristics (maternal education, household income, child age at the age 4 visit, maternal age at the time of the child’s birth, child sex assigned at birth). Children missing the inhibited temperament measure were younger (*M* = 49.44 months, *SD* = 3.90 months) than children with it (*M* = 52.33 months, *SD* = 3.31 months, *t*[116] = -2.92, *p* =.004; Cohen’s *d* = -0.86) at the age 4 assessment. Children missing baseline RSA were reported to be more socially anxious at school age (*M* = 1.47, *SD* = 0.92), compared to children with it (*M* = 0.82, *SD* = 0.68; *t*[35.18] = 3.17, *p* =.002; Cohen’s *d* = 0.85). Children missing RSA from the speech task were also reported to be more socially anxious (*M* = 1.26, *SD* = 0.87) compared to children with speech task RSA (*M* = 0.79, *SD* = 0.69; *t*[73.64] = 2.78, *p* =.007; Cohen’s *d* = 0.60). Considering primary and demographic variables, Little’s missing completely at random (MCAR) test suggested that the observed pattern of missingness did not significantly deviate from a MCAR pattern (χ^2^[174] = 192.83, *p* =.156). Missing values were handled in primary analyses by using Full Information Maximum Likelihood (FIML) estimation. Child age at the age 4 assessment acted as an auxiliary variable to aid in estimation. One participant could not be included in the speech model because they were missing all values. Thus, the sample size was 119 for the baseline model and 118 for the speech model.

### Moderation Analyses Predicting Social Anxiety Symptoms

We ran two regression models, each predicting social anxiety symptoms in M*plus* version 7.3 (Muthén & Muthén, 1998–2014); the first included baseline RSA, and the second included speech RSA. Models were just identified and thus perfectly fit; for this reason, *R*^2^ values, but not model fit statistics, are reported. The relevant RSA variable and effortful control were centered at their means prior to creating an interaction. The squared terms of the mean-centered RSA variable and effortful control were also included in accordance with previously established curvilinear relations between RSA and internalizing-domain outcomes (Acland et al., 2019; Ugarte et al., 2021) and to isolate interactive from curvilinear effects (Cohen et al., 2003). Inhibited temperament (mean-centered) was included to parse unique effects from earlier risk for social anxiety. Thus, each model included inhibited temperament, effortful control, an RSA variable, the effortful control X RSA interaction, and the quadratic terms for the RSA variable and effortful control. Model testing proceeded by testing all aforementioned terms simultaneously and dropping or probing the interaction as appropriate (Cohen et al., 2003). Significant interactions were probed by examining the simple slope of the appropriate RSA variable at the mean and ± 1 SD of effortful control. The region of significance was calculated using the Johnson-Neyman procedure. Effect sizes are indicated by *R*^2^ values for overall models (small =.01, medium =.09, large =.26) and standardized regression coefficients (*β*) for individual terms (small =.10, medium =.30, large =.50; Cohen et al., 2003).

#### Baseline RSA Model

**Fig. 1:**
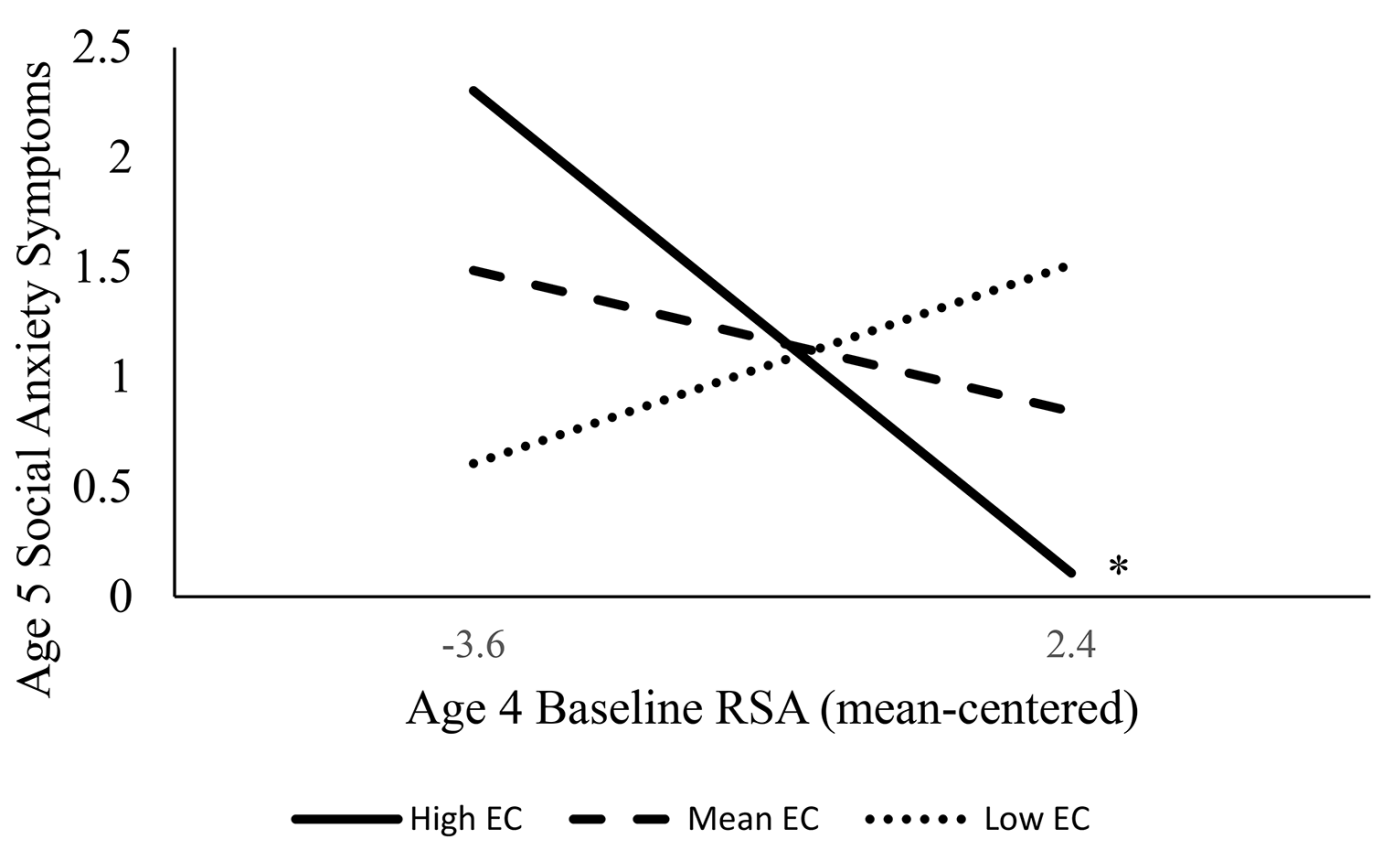
Probing of baseline RSA by effortful control interaction. Note: RSA = respiratory sinus arrhythmia. EC = effortful control. “High” EC represents + 1 SD, “Low” EC represents − 1 SD. *p <.05.

Full model results for the model including baseline RSA are presented in Table 2. The model yielded a medium effect size (*R*^*2*^ =.23, *p* =.025). The interaction between RSA and effortful control was significant and of small-to-medium effect size (*β* = -0.28). Probing of this interaction (Fig. 1) revealed that baseline RSA did not predict social anxiety symptoms at low (*b* = 0.15, *SE* = 0.15, *β* = 0.20; *t* = 1.04, *p* =.297) or mean (*b* = -0.11, *SE* = 0.09, *β* = -0.14, *t* = -1.15, *p* =.251) effortful control, but it had a negative predictive relation of medium effect size at high effortful control (*b* = -0.37, *SE* = 0.16, *β* = -0.49, *t* = -2.29, *p* =.022). The region of significance revealed that the relation shifted to significance around a value of 0.40 on the mean-centered effortful control variable, which is 0.59 SD above the mean. See Figure S1 in the Supplementary Information for the Johnson-Neyman plot. Thus, only when children were high in effortful control did lower baseline RSA predict higher social anxiety symptoms, above and beyond what would be predicted by inhibited temperament.

**Table 2:** Regression Models Predicting Age 5 Social Anxiety

	Baseline RSA						Speech RSA				
Variable	*b* (*SE*)	*β*	*t*	*p*	95% CI (*b*)		*b* (*SE*)	*β*	*t*	*p*	95% CI (*b*)
Intercept	1.10 (0.12)	--	9.33	<.001	0.87, 1.33		0.76 (0.14)	--	5.49	<.001	0.49, 1.03
Inhibited temperament	0.56 (0.15)	0.39	3.78	<.001	0.27, 0.84		0.43 (0.15)	0.30	2.88	.004	0.14, 0.72
EC	-0.13 (0.15)	-0.10	-0.84	.399	-0.42, 0.17		-0.03 (0.14)	-0.03	-0.21	.835	-0.31, 0.25
EC squared	-0.07 (0.11)	-0.08	-0.59	.558	-0.28, 0.15		0.17 (0.12)	0.20	1.45	.148	-0.06, 0.40
RSA	-0.11 (0.09)	-0.14	-1.15	.251	-0.29, 0.08		-0.40 (0.22)	-0.27	-1.81	.071	-0.83, 0.03
RSA squared	-0.07 (0.05)	-0.15	-1.31	.190	-0.18, 0.04		0.41 (0.25)	0.23	1.65	.100	-0.08, 0.90
RSA X EC	-0.38 (0.18)	-0.28	-2.14	.033	-0.73, -0.03		0.61 (0.32)	0.28	1.93	.053	-0.01, 1.24

Note. EC = Effortful Control. All terms represent mean-centered variables or the higher-order product of mean-centered variables. The Baseline model was significant (*R*^*2*^ =.23, *p* =.025). Speech RSA was residualized on Baseline RSA prior to the analysis. The Speech model was also significant (*R*^*2*^ =.26, *p* =.024).

#### Speech RSA Model

Full model results are presented in Table 2. The model explained 26% (large effect size) of the variance in social anxiety symptoms. The interaction was not significant; it had a small-to-medium effect size (*β* = 0.26). The model was rerun without the interaction to investigate main effects. The main effects model (*R*^2^ =.18, *p* =.069) revealed no significant main effects except for inhibited temperament, which was of medium effect size (*β* = 0.33).

#### Post-Hoc Exploratory Models

Because the interaction between speech RSA and effortful control approached significance (*p* =.053), we probed the interaction to provide additional information about the pattern of simple slopes for future studies with larger samples. Speech RSA had a significant negative relation to social anxiety symptoms of large effect size at low effortful control (*b* = -0.81, *SE* = 0.31, *β* = -0.56, *t* = -2.62, *p* =.009), but this relation weakened and became non-significant across mean (*b* = -0.40, *SE* = 0.22, *β* = -0.27, *t* = -1.81, *p* =.071) and high values of effortful control (*b* = 0.02, *SE* = 0.31, *β* = 0.01, *t* = 0.05, *p* =.960). The region of significance occurred at a value of -0.20 on the mean-centered effortful control variable, which was 0.30 SD below the mean (see Figure S2 in the Supplementary Information for the Johnson-Neyman plot). Thus, when children were reported to have low effortful control, lower values of speech RSA (i.e., greater suppression of RSA from baseline) predicted higher social anxiety symptoms.

We examined baseline and speech models with each of the four subscales of effortful control: attentional focusing, inhibitory control, low-intensity pleasure, and perceptual sensitivity (Tables S2 and S3 in the Supplementary Information). In the baseline RSA model, the interaction was in the same direction but weaker for attentional focusing (*β* = -0.12) and inhibitory control (*β* = -0.17), strongest and most similar to the primary model for low-intensity pleasure (*β* = -0.33), and much weaker (close to zero) for perceptual sensitivity (*β* = 0.02). In the speech RSA model, the interaction was similarly strong for attentional focusing (*β* = 0.29) and inhibitory control (*β* = 0.28), weaker but in the same direction for low-intensity pleasure (*β* = 0.12), and much weaker (close to zero) for perceptual sensitivity (*β* = 0.01).

Finally, we substituted RMSSD for RSA in the primary models (Table S4 in the Supplementary Information). Models yielded interactions in the same direction. The interaction was somewhat weaker for the baseline model but stronger and significant for the speech model.

## Discussion

Previous theory and empirical work have connected emotion dysregulation to child social anxiety development. It is becoming increasingly evident that the role of psychophysiological indicators of emotion dysregulation, such as RSA, may be more nuanced than a direct, linear relation (Mendes, 2016; Ugarte et al., 2021). To this end, we examined whether effortful control, an aspect of temperament linked to regulation (Eisenberg et al., 2014; Eisenberg & Morris, 2002), contextualized the relation between RSA and early social anxiety symptoms, with nonlinear relations specifically modeled. Controlling for inhibited temperament in earlier childhood allowed for prediction of social anxiety symptoms above and beyond what would be predicted from a robust predictor of risk, as well as differentiation of RSA and effortful control from reactive control (Eisenberg et al., 2014; Eisenberg & Morris, 2002).

Resting RSA did have the expected negative relation to anxiety, similarly to previous studies (Sharma et al., 2011), but, contrary to our hypothesis, this association emerged at high, rather than low, effortful control. We expected that RSA would relate to social anxiety symptoms for children who were reported to have lower top-down control over behavior. However, RSA did not show any relation to social anxiety symptoms at low effortful control. Relatively lower RSA at rest may indicate inflexibility in one’s capacity to respond to the environment. Thus, inflexibility in the context of higher effortful control may reflect discordance among multiple regulatory systems, resulting in dysregulated negative emotionality that poses risk for anxiety (Hassan et al., 2018). Although effortful control is separable from reactive control (Eisenberg et al., 2014; Eisenberg & Morris, 2002), this distinction may be less clear in regards to anxiety risk. Our findings may align with research showing that high inhibitory control, a subdomain of effortful control, characterizes automatic or involuntary control efforts that facilitate, rather than decrease, social anxiety risk for children who are already temperamentally prone to anxious outcomes (Fox et al., 2023). However, sensitivity analyses in our study did not suggest that inhibitory control functioned in a unique manner from other subdomains of effortful control. Interpreting this relation in another way, children high in effortful control and high resting RSA appeared to be at decreased risk for anxiety. In the context of high effortful control, greater RSA may allow for flexibility in regulation. We encourage others to attempt to replicate these results.

Conclusions related to task-related changes in RSA are more tentative. The speech task elicited a range of individual differences in distress and shyness, with a sizeable portion of the sample showing moderate to extreme behaviors relevant to anxiety. In an exploratory model, when children were reported to be low in effortful control, RSA that was lower than expected given baseline values (i.e., more negative residuals) predicted greater social anxiety. This suggests greater suppression of the parasympathetic response in a socially evaluative situation, or a “threat” response, with children’s resources (e.g., effortful control capacity) falling short of the demands of the situation. In contrast, children low in resources (i.e., low effortful control) whose parasympathetic system did not mount a threat response showed relatively lower social anxiety symptoms. This relation between RSA suppression and social anxiety risk is similar to a previous study that also used a speech task as a stressor (Poole & Schmidt, 2021). Our work extends these findings to show that children whose psychophysiology mounts a threat response may be a specific subgroup who are low in effortful control. Although these results should be interpreted with caution, they do suggest continued investigation into how temperament contextualizes the implications of task-related psychophysiology.

Effortful control is a multidimensional construct, comprising capacities for holding back dominant responses, orienting and shifting attention, and experiencing pleasure while engaging in low arousal activities (Eisenberg et al., 2014). Although these subdomains converge for children, generally, they may be differentially related to anxiety risk for temperamentally inhibited children, specifically. Fox et al.’s (2023) Detection and Dual Control (DDC) framework delineates how automatic (inhibitory control) and planful (attention-shifting) regulation strengthen versus disrupt stability from inhibited temperament to anxiety-based outcomes. We did not find unique patterns of results for the inhibitory control versus attention domains of effortful control in this sample; however, we did not have a measure of attention-shifting, specifically. Although we controlled for inhibited temperament, we did not examine it as an additional moderator in our model, both for conceptual simplicity and in acknowledgement of our limited statistical power for detecting three-way interactions. With a larger sample, it may have been informative to test whether, beyond being considered a covariate, inhibited temperament further contextualizes the interactions between effortful control and RSA, and whether these interactions are different across automatic versus planful subdomains, as predicted by the DDC framework (Fox et al., 2023). For example, it would be helpful to know whether interactive effects of regulatory systems are especially strong for very inhibited children, and whether inhibitory control plays a unique role in prediction of risk. Alternatively, perhaps interactions among aspects of regulation are especially meaningful for uninhibited children, who do not have existing risk for anxiety. In other words, multisystem dysregulation may be an alternative pathway to anxiety for children who are not characterized as inhibited.

Regulatory systems continue to develop across and beyond the age range represented in our sample. Significant development in the neural systems underlying regulation has already occurred by age 4 (Eisenberg et al., 2014), which may have been reflected in the strong stability of effortful control to our follow up assessment. As children approach age 8 and beyond, their increased understanding of emotions allows for increasingly cognitive strategies of regulation that may affect both their psychophysiology and behavior (Eisenberg & Morris, 2002). The current study offers insight about the relation of emotion-related regulation to anxiety risk in the preschool/early school age period, but these relations may change as children develop.

Clinically, our results support current interventions that focus on increasing children’s regulatory capacities in an effort to decrease current or future experiences of anxiety. For example, the Unified Protocol for Transdiagnostic Treatment of Emotional Disorders in Children (Ehrenreich-May et al., 2018) builds skills in cognitive reappraisal and other coping strategies for managing intense emotional reactions. However, children with social anxiety may not experience as high of treatment gains as children with other presenting diagnoses when receiving psychotherapy alone, perhaps benefitting from psychophysiological intervention (i.e., medication) in addition to cognitive-behavioral psychotherapy (Compton et al., 2014). Our results support the idea that both psychophysiological and more cognitively accessible aspects of regulation are relevant for children showing social anxiety symptoms at early school age, predating when children typically start treatment. Prevention programs targeting this age group, such as Cool Little Kids (Bayer et al., 2021), may reduce anxiety symptoms prior to reaching diagnostic levels. Testing whether early intervention changes RSA for children at varying levels of effortful control would augment theory of the role of emotion dysregulation in social anxiety etiology, and also provide further rationale for early preventative intervention for social anxiety.

Results should be considered in light of the limitations associated with this study. As mentioned, the moderate sample size, although appropriate for testing hypotheses herein, may have prevented the detection of interactive effects and precluded analysis of more complex statistical models. The sample is most representative of White, middle-class families in the Midwestern United States. Culture and other contextual factors play a significant role in shaping the values and socialization of self-regulation (Raval & Walker, 2019). By preschool age, children’s development has been shaped by their surroundings in a manner that reinforces and punishes particular behaviors over others. Thus, whether particular behavioral and psychophysiological patterns conform to environmental expectations to predict adaptive outcomes, or contrast with typical expectations and thus promote anxiety, depends on the culture and context with which children dynamically interact as they develop. As such, we caution against generalizing our results beyond the population represented by our sample, which was largely shaped by values of independence and maximization of positive emotions, characteristic of families living in Western, predominantly White, and socioeconomically advantaged contexts. We did not consider how maternal behavior during the speech may have affected children’s psychophysiological responding, which is an important future direction for understanding the multiple influences on children’s regulation. Finally, mothers reported both effortful control and social anxiety symptoms. Although moderation analyses did not depend on the relation between these variables, they nevertheless share any biases or filters inherent in parent report.

## Conclusion

The current study lends support to current theory that psychophysiological and behavioral indicators of emotion dysregulation may work interactively, rather than simply additively or interchangeably, in the etiology of child social anxiety symptoms. The nature by which effortful control contextualized psychophysiological risk depended on whether baseline or social stressor tasks were considered. Low resting, and greater suppression of, respiratory sinus arrhythmia, predicted greater social anxiety symptoms a year later in the context of high and low effortful control, respectively. Unique from the pathway between inhibited temperament and social anxiety, then, multi-system emotion dysregulation is an important component of etiological risk.

## Electronic Supplementary Material

Below is the link to the electronic supplementary material.

Supplementary Material 1
