# Supplementary material for: Respiratory Sinus Arrhythmia, Effortful Control, and Child Social Anxiety Symptoms
Source: Res Child Adolesc Psychopathol. 2024 Apr 26;52(9):1441–53. doi: 10.1007/s10802-024-01202-z (PMC11420266; doi:10.1007/s10802-024-01202-z)
Supplement: Supplementary file 1 — Supplementary Material 1 [file 10802_2024_1202_MOESM1_ESM.docx]

**Respiratory Sinus Arrhythmia, Effortful Control, and Children’s Social Anxiety Symptoms**

Supplementary Information

Introduction to the Supplement

This supplement contains several pieces of information referenced in the main text of the manuscript. Data and code from these analyses are available from the first author.

First, we re-analyzed our primary models using RSA variables derived from a 0.33 to 0.50 frequency band (Table S1) to compare to our reported results with RSA derived from the 0.24 to 1.04 frequency band. These new RSA values were highly correlated with our original baseline (*r* = .98) and speech (*r* = .97) values. Compared to our original baseline (*M* = 5.10, *SD* = 1.09) and speech (*M* = 5.04, *SD* = 1.08) RSA scores, scores from the .33 to .50 frequency band were significantly lower for baseline (*M* = 3.70, *SD* = 1.14; *t* = 57.43, *p* < .001; Cohen’s *d* = 6.05) and speech (*M* = 3.53, *SD* = 1.15; *t* = 43.49, *p* < .001; Cohen’s *d* = 5.24). When we regressed speech on baseline to get the residualized value analyzed as RSA change, theses scores were also highly (although not AS highly) correlated (*r* = .88, *p* < .001). Results of primary models were highly similar to those reported in the manuscript in terms of direction and strength of effects.

Next, we re-ran our models using each of the subdomains of effortful control (attentional focusing, inhibitory control, low-intensity pleasure, and perceptual sensitivity). The results of these models are summarized in Tables S2 and S3.

We also re-ran primary models using RMSSD instead of RSA. Baseline RMSSD represented the average of RMSSD across the five 1-minute segments of the baseline period. Values ranged from 4.38 to 96.52 (*M* = 27.96, *SD* = 16.16). RMSSD was recorded for each of the 3 minutes of the speech task, and these values were averaged (*M* = 27.87, *SD* = 19.73, *range* = 5.31 to 144.82). RMSSD from the speech task demonstrated deviation from normality (skew = 3.45, kurtosis = 17.72). To be consistent across variables, we applied a square root transformation to both baseline RMSSD (*M* = 5.10, *SD* = 1.42, *range* = 2.09 to 9.82) and speech RMSSD (*M* = 5.06, *SD* = 1.52, *range* = 2.31 to 12.03), which resulted in more typical values of skew (1.68) and kurtosis (5.55) for the speech average. The final “speech RMSSD” variable represented the unstandardized residual from the regression of speech RMSSD on baseline RMSSD. RMSSD was highly correlated with our original RSA values for baseline (*r* = .95) and speech (residualized on baseline, *r* = .79). In the baseline model (Table S4), the interaction term was weaker and fell to non-significance, but it remained in the same direction and of small to medium effect size. In the speech model (Table S4), the interaction term was stronger (medium effect size) and was significant, in the same direction as reported in the manuscript.

| **Table S1** |  |  |  |  |  |  |  |  |  |  |  |
| --- | --- | --- | --- | --- | --- | --- | --- | --- | --- | --- | --- |
| *Regression Models Predicting Age 5 Social Anxiety Using 0.33 to 0.50 Frequency Band for RSA* | | | | | | | | | | | |
|  |  |  |  |  |  |  |  |  |  |  |  |
|  |  |  |  |  |  |  |  |  |  |  |  |
|  | Baseline RSA | | | | |  | Speech RSA | | | | |
|  |  |  |  |  |  |  |  |  |  |  |  |
| Variable | *b* (*SE*) | *β* | *t* | *p* | 95% CI (*b*) |  | *b* (*SE*) | *β* | *t* | *p* | 95% CI (*b*) |
|  |  |  |  |  |  |  |  |  |  |  |  |
|  |  |  |  |  |  |  |  |  |  |  |  |
| Intercept | 1.10 (0.12) | -- | 9.42 | <.001 | 0.88, 1.34 |  | 0.87 (0.13) | -- | 6.95 | <.001 |  |
|  |  |  |  |  |  |  |  |  |  |  |  |
| Inhibited temperament | 0.57 (0.15) | 0.40 | 3.83 | <.001 | 0.28, 0.86 |  | 0.37 (0.15) | 0.26 | 2.45 | .014 | 0.07, 0.67 |
|  |  |  |  |  |  |  |  |  |  |  |  |
| EC | -0.15 (0.15) | -0.13 | -1.04 | .297 | -0.45, 0.14 |  | 0.01 (0.14) | 0.01 | 0.06 | .953 | -0.36, 0.29 |
|  |  |  |  |  |  |  |  |  |  |  |  |
| EC squared | -0.10 (0.12) | -0.11 | -0.85 | .394 | -0.33, 0.13 |  | 0.11 (0.11) | 0.13 | 1.01 | .312 | -0.10, 0.33 |
|  |  |  |  |  |  |  |  |  |  |  |  |
| RSA | -0.12 (0.09) | -0.17 | -1.33 | .184 | -0.30, 0.06 |  | -0.17 (0.21) | -0.13 | -0.82 | .410 | -0.57, 0.23 |
|  |  |  |  |  |  |  |  |  |  |  |  |
| RSA squared | -0.07 (0.05) | -0.16 | -1.39 | .164 | -0.17, 0.03 |  | 0.11 (0.15) | 0.11 | 0.75 | .451 | -0.18, 0.40 |
|  |  |  |  |  |  |  |  |  |  |  |  |
| RSA X EC | -0.39 (0.17) | -0.30 | -2.33 | .020 | -0.72, -0.06 |  | 0.65 (0.37) | 0.27 | 1.75 | .079 | -0.08, 1.37 |
|  |  |  |  |  |  |  |  |  |  |  |  |
| *Note*. EC = Effortful Control. All terms represent mean-centered variables or the higher-order product of mean-centered variables. The Baseline model was significant (*R^2^* = .24, *p* = .021). Speech RSA was residualized on Baseline RSA prior to the analysis. The Speech model was marginally significant (*R^2^* = .18, *p* = .058). | | | | | | | | | | | |

| **Table S2** |  |  |  |  |  |  |  |  |  |  |  |
| --- | --- | --- | --- | --- | --- | --- | --- | --- | --- | --- | --- |
| *Baseline RSA Model Results for Specific Domains of Effortful Control* | | | | | |  |  |  |  |  |  |
|  |  |  |  |  |  |  |  |  |  |  |  |
|  |  |  |  |  |  |  |  |  |  |  |  |
|  | Attentional Focusing | |  | Inhibitory Control | |  | Low Intensity Pleasure | |  | Perceptual Sensitivity | |
|  |  |  |  |  |  |  |  |  |  |  |  |
| Variable | *β* | *p* |  | *β* | *p* |  | *β* | *p* |  | *β* | *p* |
|  |  |  |  |  |  |  |  |  |  |  |  |
|  |  |  |  |  |  |  |  |  |  |  |  |
| Inhibited temperament | 0.37 | <.001 |  | 0.38 | <.001 |  | 0.40 | <.001 |  | 0.33 | .001 |
|  |  |  |  |  |  |  |  |  |  |  |  |
| EC subscale | -0.07 | .517 |  | -0.13 | .227 |  | -0.04 | .736 |  | 0.09 | .501 |
|  |  |  |  |  |  |  |  |  |  |  |  |
| EC subscale squared | 0.04 | .712 |  | -0.12 | .309 |  | 0.09 | .566 |  | 0.11 | .378 |
|  |  |  |  |  |  |  |  |  |  |  |  |
| Baseline RSA | -0.09 | .491 |  | -0.13 | .316 |  | -0.13 | .317 |  | -0.09 | .495 |
|  |  |  |  |  |  |  |  |  |  |  |  |
| Baseline RSA squared | -0.15 | .204 |  | -0.22 | .070 |  | 0.01 | .960 |  | -0.15 | .264 |
|  |  |  |  |  |  |  |  |  |  |  |  |
| Baseline RSA X EC subscale | -0.12 | .332 |  | -0.17 | .199 |  | -0.33 | .016 |  | 0.02 | .910 |
|  |  |  |  |  |  |  |  |  |  |  |  |
| *Note*. EC = effortful control. All terms represent mean-centered variables or the higher-order product of mean-centered variables. | | | | | | | | | | | |

| **Table S3** |  |  |  |  |  |  |  |  |  |  |  |
| --- | --- | --- | --- | --- | --- | --- | --- | --- | --- | --- | --- |
| *Speech RSA Model Results for Specific Domains of Effortful Control* | | | | | |  |  |  |  |  |  |
|  |  |  |  |  |  |  |  |  |  |  |  |
|  |  |  |  |  |  |  |  |  |  |  |  |
|  | Attentional Focusing | |  | Inhibitory Control | |  | Low Intensity Pleasure | |  | Perceptual Sensitivity | |
|  |  |  |  |  |  |  |  |  |  |  |  |
| Variable | *β* | *p* |  | *β* | *p* |  | *β* | *p* |  | *β* | *p* |
|  |  |  |  |  |  |  |  |  |  |  |  |
|  |  |  |  |  |  |  |  |  |  |  |  |
| Inhibited temperament | 0.31 | .003 |  | 0.34 | .001 |  | 0.31 | .002 |  | 0.29 | .005 |
|  |  |  |  |  |  |  |  |  |  |  |  |
| EC subscale | -0.07 | .597 |  | -0.15 | .175 |  | -0.02 | .883 |  | 0.11 | .370 |
|  |  |  |  |  |  |  |  |  |  |  |  |
| EC subscale squared | 0.09 | .421 |  | -0.01 | .939 |  | 0.18 | .296 |  | 0.17 | .208 |
|  |  |  |  |  |  |  |  |  |  |  |  |
| Speech RSA | -0.22 | .175 |  | -0.19 | .224 |  | -0.28 | .067 |  | -0.26 | .100 |
|  |  |  |  |  |  |  |  |  |  |  |  |
| Speech RSA squared | 0.10 | .550 |  | 0.26 | .072 |  | 0.27 | .056 |  | 0.25 | .086 |
|  |  |  |  |  |  |  |  |  |  |  |  |
| Speech RSA X EC subscale | 0.29 | .064 |  | 0.28 | .041 |  | 0.12 | .474 |  | 0.01 | .972 |
|  |  |  |  |  |  |  |  |  |  |  |  |
| *Note*. EC = effortful control. All terms represent mean-centered variables or the higher-order product of mean-centered variables. | | | | | | | | | | | |

| **Table S4** |  |  |  |  |  |  |  |  |  |  |  |
| --- | --- | --- | --- | --- | --- | --- | --- | --- | --- | --- | --- |
| *Regression Models Predicting Age 5 Social Anxiety Using RMSSD* | | | | | | | | | | | |
|  |  |  |  |  |  |  |  |  |  |  |  |
|  |  |  |  |  |  |  |  |  |  |  |  |
|  | Baseline RMSSD | | | | |  | Speech RMSSD | | | | |
|  |  |  |  |  |  |  |  |  |  |  |  |
| Variable | *b* (*SE*) | *β* | *t* | *p* | 95% CI (*b*) |  | *b* (*SE*) | *β* | *t* | *p* | 95% CI (*b*) |
|  |  |  |  |  |  |  |  |  |  |  |  |
|  |  |  |  |  |  |  |  |  |  |  |  |
| Intercept | 1.06 (0.12) | -- | 8.62 | <.001 | 0.82, 1.30 |  | 0.92 (0.11) | -- | 8.16 | <.001 | 0.70, 1.14 |
|  |  |  |  |  |  |  |  |  |  |  |  |
| Inhibited temperament | 0.53 (0.16) | 0.38 | 3.40 | .001 | 0.23, 0.84 |  | 0.37 (0.15) | 0.26 | 2.43 | .015 | 0.07, 0.66 |
|  |  |  |  |  |  |  |  |  |  |  |  |
| EC | -0.10 (0.15) | -0.08 | -0.63 | .526 | -0.39, 0.20 |  | -0.03 (0.14) | -0.02 | -0.19 | .850 | -0.31, 0.25 |
|  |  |  |  |  |  |  |  |  |  |  |  |
| EC squared | -0.05 (0.12) | -0.05 | -0.39 | .699 | -0.28, 0.19 |  | 0.09 (0.11) | 0.10 | 0.83 | .404 | -0.12, 0.29 |
|  |  |  |  |  |  |  |  |  |  |  |  |
| RMSSD | -0.04 (0.08) | -0.07 | -0.49 | .626 | -0.19, 0.12 |  | -0.18 (0.16) | -0.16 | -1.11 | .267 | -0.49, 0.14 |
|  |  |  |  |  |  |  |  |  |  |  |  |
| RMSSD squared | -0.03 (0.03) | -0.11 | -0.81 | .421 | -0.09, 0.04 |  | 0.05 (0.09) | 0.09 | 0.61 | .542 | -0.12, 0.23 |
|  |  |  |  |  |  |  |  |  |  |  |  |
| RMSSD X EC | -0.23 (0.15) | -0.22 | -1.55 | .121 | -0.52, 0.06 |  | 0.62 (0.29) | 0.33 | 2.10 | .036 | 0.04, 1.19 |
|  |  |  |  |  |  |  |  |  |  |  |  |
| *Note*. RMSSD = Root Mean Square Successive Difference. EC = Effortful Control. A square root transformation was applied to both baseline and speech RMSSD. All terms represent mean-centered variables or the higher-order product of mean-centered variables. The Baseline model was marginally significant (*R^2^* = .17, *p* = .073). Speech RMSSD was residualized on Baseline RMSSD prior to the analysis. The Speech model was significant (*R^2^* = .19, *p* = .039). | | | | | | | | | | | |

**Figure S1**

Johnson-Neyman Plot for Baseline RSA Model


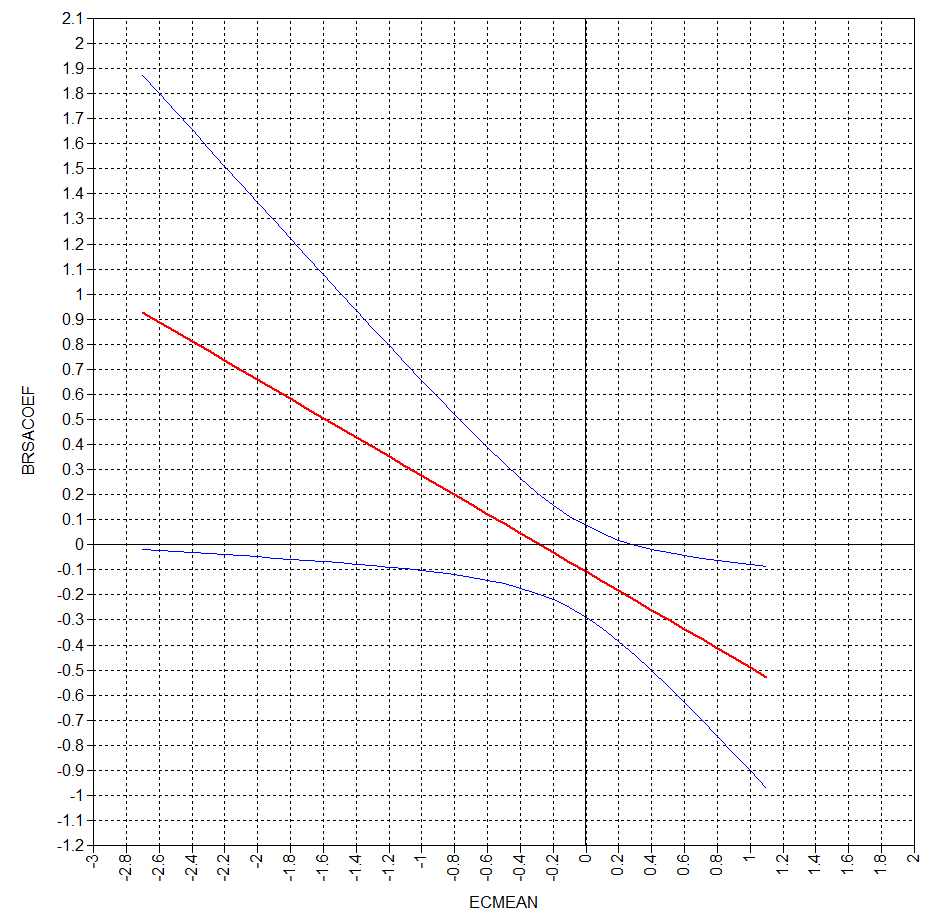


*Note*. Plot represents the interaction between effortful control centered at its mean (ECMEAN) and baseline RSA (BRSACOEF) in relation to child social anxiety symptoms. The red line represents the relation between the moderator (ECMEAN) and the coefficient attached to baseline RSA (BRSACOEF). The blue lines represent the 95% confidence interval surrounding the coefficient. The coefficient is significant when the blue lines do not contain zero, here at values ≥ 0.40 of mean-centered effortful control, which is about 0.59 SD above the mean.

**Figure S2**

Johnson-Neyman Plot for Speech RSA Model.


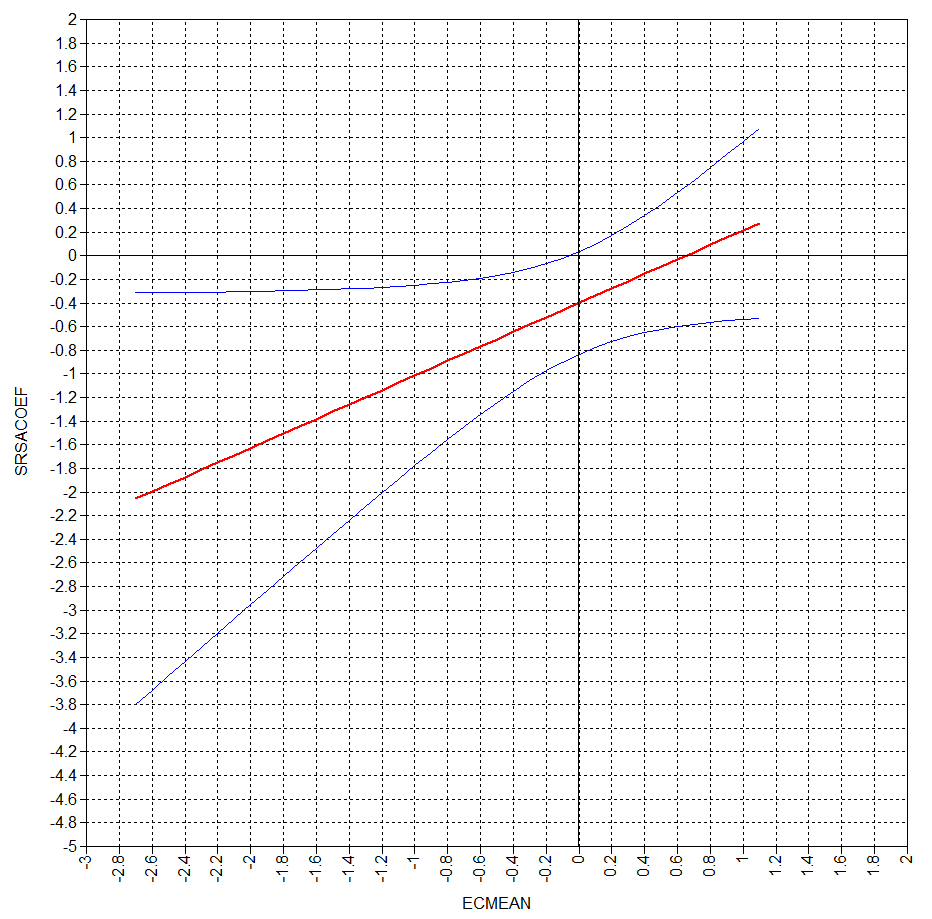


Note. Plot represents the interaction between effortful control centered at its mean (ECMEAN) and speech RSA (SRSACOEF) in relation to child social anxiety symptoms. The red line represents the relation between the moderator (ECMEAN) and the coefficient attached to speech RSA (SRSACOEF). The blue lines represent the 95% confidence interval surrounding the coefficient. The coefficient is significant when the blue lines do not contain zero, here at values ≤ -0.20 on mean-centered effortful control, which is about 0.30 SD below the mean.
